# Utilization of RT-PCR and Optical Genome Mapping in Acute Promyelocytic Leukemia with Cryptic *PML::RARA* Rearrangement: A Case Discussion and Systemic Literature Review

**DOI:** 10.3390/genes16010007

**Published:** 2024-12-25

**Authors:** Giby V. George, Murad Elsadawi, Andrew G. Evans, Sarmad Ali, Bin Zhang, M. Anwar Iqbal

**Affiliations:** 1 Department of Pathology and Laboratory Medicine, University of Rochester Medical Center, Rochester, NY 14642, USAsarmad_ali@urmc.rochester.edu (S.A.);; 2 Department of Pathology, University of Pittsburgh Medical Center, Pittsburgh, PA 15213, USA

**Keywords:** acute promyelocytic leukemia, APL, cryptic *PML::RARA*, cryptic APL

## Abstract

Background: Acute promyelocytic leukemia (APL) is characterized by abnormal promyelocytes and t(15;17)(q24;q21) *PML::RARA*. Rarely, patients may have cryptic or variant rearrangements. All-trans retinoic acid (ATRA)/arsenic trioxide (ATO) is largely curative provided that the diagnosis is established early. Methods: We present the case of a 36-year-old male who presented with features concerning for disseminated intravascular coagulation. Although the initial diagnostic work-up, including pathology and flow cytometry evaluation, suggested a diagnosis of APL, karyotype and fluorescence in situ hybridization (FISH), using the *PML/RARA* dual fusion and *RARA* breakapart probes, were negative. We performed real-time polymerase chain reaction (RT-PCR) and optical genome mapping (OGM) to further confirm the clinicopathological findings. Results: RT-PCR revealed a cryptic *PML::RARA* fusion transcript. OGM further confirmed the nature and orientation of a cryptic rearrangement with an insertion of *RARA* into *PML* at intron 3 (bcr3). In light of these findings, we performed a systematic literature review to understand the prevalence, diagnosis, and prognosis of APL with cryptic *PML::RARA* rearrangements. Conclusions: This case, in conjunction with the results of our systematic literature review, highlights the importance of performing confirmatory testing in FISH-negative cases of suspected APL to enable prompt diagnosis and appropriate treatment.

## 1. Introduction

Acute promyelocytic leukemia (APL) is a subtype of acute myeloid leukemia (AML) that accounts for 10–15% of all new AML diagnoses [1]. Patients present with features of coagulopathy and are at increased risk of disseminated intravascular coagulation (DIC) [2]. Morphologically, APL is characterized by abundant abnormal promyelocytes with a classic immunophenotype demonstrable by flow cytometry [2]. Per the WHO classification, the diagnosis of APL is rendered based on these microscopic findings in conjunction with t(15;17)(q24.1;q21.2) [2]. This rearrangement results in the fusion of the 5′ end of the promyelocytic leukemia (*PML*) gene (exons 1–3) on chromosome 15 with the 3′ end of the retinoic acid receptor α (*RARA*) gene (exons 3–9) on chromosome 17 [2,3,4]. The International Consensus Classification of Myeloid Neoplasms and Acute Leukemias (ICC) requires at least 10% blasts (or blast equivalents, i.e., abnormal promyelocytes) for diagnosis [3]. Morphologic subtypes of APL include the hypergranular (i.e., typical) and hypogranular (i.e., microgranular) forms (with a basophilic variant described in some studies) [2,3,4]. Notably, most APL subtypes, including cases with variant *RARA* translocations, demonstrate similar morphologies [3]. Intriguingly, APL-like morphologies have been observed in other genetic subtypes of AML, most notoriously *NPM1*-mutated AML; therefore, thorough cytogenetic and molecular workup is needed to confirm the diagnosis [5,6]. 

The t(15;17)(q24.1;q21.2) translocation can usually be identified by fluorescence in situ hybridization (FISH) using *PML/RARA* dual fusion or *RARA* breakapart probes or chromosomal Giemsa (G)-banding analysis. The reciprocal t(15;17) translocation is present in 90–95% of APL cases, and, depending on the location of the *PML* breakpoint, three *PML::RARA* fusion transcript isoforms can be produced: long (bcr1; intron 6), variant (bcr2; exon 6), and short (bcr3; intron 3), with the bcr1 and bcr3 isoforms being the most common [2,7,8]. Rarely, atypical isoforms can be also be detected [2]. The *RARA* breakpoint consistently occurs within intron 2 [7]. A subset of APL cases (~5–9%) [9] may show cryptic *PML::RARA* rearrangement with submicroscopic insertion of *PML* to *RARA,* or complex rearrangements involving other chromosomes. Other cases of APL (~1–2%) [10] may show variant *RARA* rearrangements [2,4,11,12,13,14,15,16]. Importantly, cases of APL with cryptic rearrangements may require alternative methods of detection [2].

Real-time PCR (RT-PCR) is a widely used molecular technique for the detection of specific genetic markers in hematological malignancies including the *PML/RARA* fusion transcript in APL [17,18,19,20]. RT-PCR has been instrumental in identifying cryptic rearrangements in APL that are not detected by standard of care cytogenetic testing due to the low resolution of karyotyping (10–15 Mbp in cancer) and/or poor performance of FISH in atypical rearrangements due to specific probe design [7,21]. RT-PCR is the preferred tool for monitoring minimal residual disease (MRD) due to its high sensitivity, capable of detecting levels as low as 1 in 10,000 to 100,000 cells [22].

OGM is a promising next-generation cytogenomics technique that has shown significant potential in clinical cytogenetics [23,24,25,26]. OGM provides superior resolution, i.e., ~5 kb for structural variants and 250–500 kb for copy number variants, which is ~1000–20,000 times higher compared to conventional cytogenetic methods, allowing for the detection of small and complex genomic alterations that are crucial for accurate diagnosis and prognosis [27].

Herein, we describe a case of cryptically rearranged APL that showed a normal karyotype and normal dual color dual fusion t(15;17) and *RARA* breakapart FISH results. RT-PCR and OGM identified a cryptic *PML::RARA* fusion caused by the insertion of 62 kb of *RARA* into the *PML* gene. Fortunately, the patient had been initiated on all-trans retinoic acid (ATRA)/arsenic trioxide (ATO) early based on the morphological/immunophenotypic findings and demonstrated clinical improvement. These findings, in conjunction with those of our systematic literature review, highlight the importance of performing confirmatory testing in FISH-negative cases of suspected APL.

## 2. Materials and Methods

### 2.1. Pathological Examination

Standard Wright Giemsa peripheral blood smears were prepared.

### 2.2. Clinical Flow Cytometry Evaluation

Expression of cell surface markers was measured by flow cytometry (Navios, Beckman Coulter, Indianapolis, IN, USA) using the ClearLLab 10C 10-color lymphoid and myeloid panels and analyzed using the Kaluza C analysis software version 1.2.

### 2.3. Chromosome G-Banding Karyotype Analysis

Cytogenetic analysis was performed using short-term bone marrow cultures per routine laboratory protocol. For microscopic analysis, metaphase chromosomes were stained using the trypsin-Giemsa technique [28]. For chromosome analysis, 20 cells were analyzed and two to five metaphases were karyotyped. Chromosomal abnormalities were defined according to ISCN (2020) [29].

### 2.4. Fluorescence In Situ Hybridization

Fluorescence in situ hybridization (FISH) was performed on 200 interphase nuclei using the AML panel probes (Abbott Molecular/Vysis, Inc., Des Plaines, IL, USA): LSI *EGR1* (5q31) SO/D5S23, D5S721 SG, LSI D7S486 (7q31) SO/CEP 7 (D7Z1) SG, LSI *RUNX1T1/RUNX1* Dual Color, Dual Fusion Translocation, LSI *KMT2A* Dual Color, Break Apart Rearrangement, LSI *PML/RARA* Dual Color, Dual Fusion Translocation, LSI *CBFB* Dual Color, Break Apart Rearrangement, LSI 13 (13q14) SG/LSI TP53 (17p13.1) SO, and LSI *RARA* Dual Color, Break Apart Rearrangement probes. Retrospective interphase FISH using the CytoCell (Oxford Gene Technology, Tarrytown, NY, USA) *PML/RARA* dual-color dual-fusion probe set was performed, followed by confirmatory metaphase FISH using the CytoCell probe. The design of the probes used are as follows: the Vysis dual color dual fusion *PML/RARA* probe consists of fluorescently labeled DNA that spans approximately 180 kb and 335 kb on either side of the *PML* loci on chromosome 15, covering all known BCR regions of *PML*, as well as about 700 kb of chromosome 17, encompassing the entire breakpoint region of *RARA*, while the CytoCell *PML/RARA* probe consists of fluorescently labeled DNA that spans approximately 151 kb and 174 kb on either side of the *PML* loci on chromosome 15, covering D15S169 and D15S965 markers and 167 kb and 164 kb on either side of the *RARA* loci on chromosome 17.

### 2.5. Mutational Analysis

*FLT3* mutational analysis was performed using rapid multiplex PCR. Targeted DNA-based NGS was performed using the 34-gene Illumina TruSight Myeloid Panel (San Diego, CA, USA).

### 2.6. Optical Genome Mapping

Optical Genome Mapping (OGM) was performed as described previously [30]. Briefly, ultra-high molecular weight DNA was isolated from the patient’s peripheral blood sample using the SP Blood and Cell Culture DNA Isolation Kit. The DLS DNA Labeling Kit was used to fluorescently label long molecules at specific CTTAAG motifs throughout the genome with the enzyme DLE-1 (Bionano Genomics, San Diego, CA, USA). Labeled DNA was loaded onto a chip and imaged on the Saphyr instrument for the collection of 1500 Gb data with a molecule size of >150 kb. Data analysis was performed using the Bionano Access Software (BAS) Version 1.8.1 and Variant Intelligence Analysis (VIA) version 7.0

### 2.7. Systematic Literature Review

A systematic literature review was conducted through a targeted search (using the following search terms: “APL with cryptic translocation”, “APL with negative FISH”, “APML with negative FISH”, and “APL with RT-PCR”) for case reports and original articles published in English journals from 2013–2023 archived in PubMed.

## 3. Results

### 3.1. Case Description

**Figure 1:**
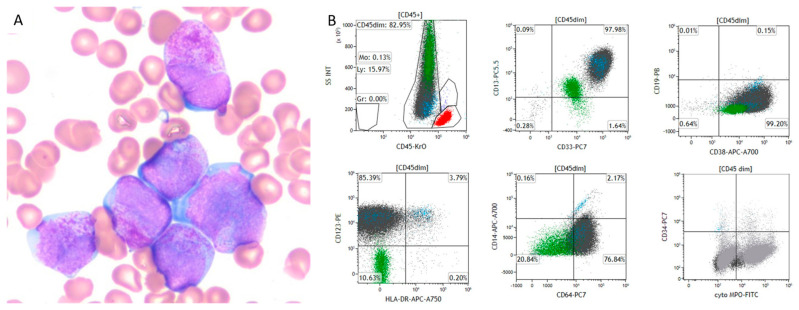
Peripheral Smear Findings. (**A**). Morphologic examination revealed scattered abnormal promyelocytes with variable cytoplasmic granules and occasional Auer rods. (**B**). Flow cytometry evaluation of the peripheral blood specimen shows an abnormal cell population sitting within the granulocytic gate (displayed in gray, over 80% of total analyzed cells). The lymphocytic gate is depicted in red. The abnormal population with high SSC shows co-expression of CD13, CD33, CD117, CD38, CD123, CD64 and cytoplasmic-MPO, while negative for CD34, HLA-DR, and all other markers tested.

A 36-year-old male presented with spontaneous ecchymosis, petechiae, and exertional dyspnea. Laboratory investigations revealed anemia (hemoglobin 11.2 g/dL), thrombocytopenia (platelets 10 × 10^3^/µL), hypofibrinogenemia (70 mg/dL), prolonged prothrombin time (14.7 s)/international normalization ratio (INR) (1.3), and elevated D-dimer (26.20 µg/mL), concerning for DIC. Peripheral blood examination demonstrated pancytopenia. Scattered abnormal promyelocytes with bilobed nuclei, occasional Auer rods, and variable cytoplasmic granules were noted by microscopy, raising concern for APL (Figure 1A). Consistent with this preliminary diagnosis, flow cytometry evaluation revealed an abnormal cell population within the CD45-dim gate with high side scatter and co-expression of CD13, CD33, CD117, CD38, CD123, CD64, and cytoplasmic myeloperoxidase (MPO), while negative for all other markers tested (Figure 1B).

### 3.2. Standard of Care Cytogenetic Findings

**Figure 2:**
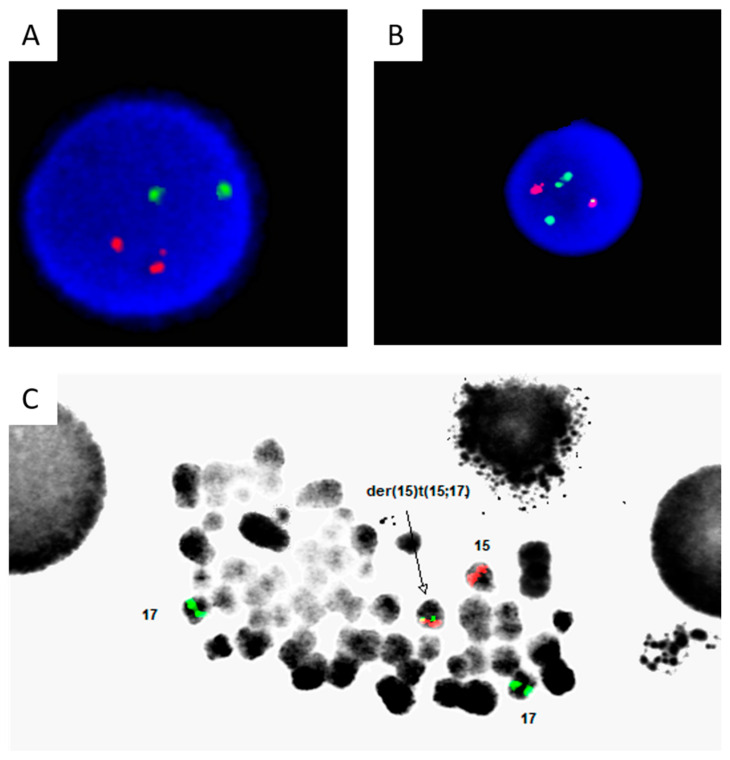
(**A**). Interphase FISH performed using the Vysis dual color dual fusion t(15;17) probe, showing normal signals for both *PML* (SpectrumOrange) and *RARA* (SpectrumGreen). (**B**). Retrospective FISH using the CytoCell *PML/RARA* dual color dual fusion probe set, which targets smaller regions, revealed a fusion in 74% of interphase cells. (**C**). Metaphase FISH also confirmed this cryptic rearrangement as depicted by the arrow.

Chromosome G-banding analysis revealed a normal male karyotype in 20 metaphases analyzed. Stat FISH using the Abbott, Vysis probe was negative for the classical t(15;17) rearrangement (Figure 2A). Retrospective FISH using the CytoCell *PML/RARA* dual color dual fusion probe revealed a fusion in 74% of interphase cells (Figure 2B). Metaphase FISH also confirmed this aberration (Figure 2C).

### 3.3. RT-PCR PML::RARA Fusion and Molecular Testing

Send-out RT-PCR, performed at Quest Diagnostics, detected a cryptic *PML::RARA* gene fusion in the form of the short (bcr3) transcript isoform. There was no evidence of a mutations in FLT3-ITD or *FLT3* codons 835/836. Targeted DNA-based NGS subsequently revealed an oncogenic mutation in *NRAS* (c.35G>T, p.G12V) at a variant allele frequency (VAF) of 11%.

### 3.4. Optical Genome Mapping Findings

**Figure 3:**
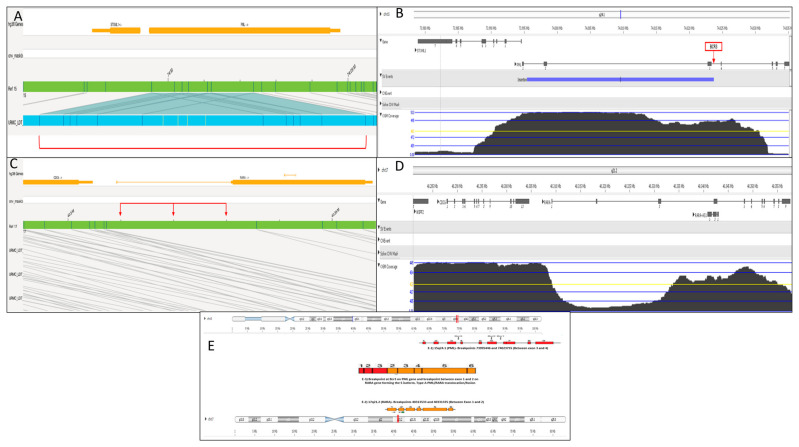
Optical Genome Mapping Results. (**A**). Genome browser view using the BAS software 1.8.1 showed an insertion in the *PML* gene at breakpoints 73995446 and 74023755 marked in red. The light blue horizontal bar represents the sample’s consensus map aligned with reference chromosome 15 map represented as a light green horizontal bar. The insertion in chromosome 15 in the middle is denoted by the gray lines. (**B**). Further analysis using the VIA software 7.0 confirmed the insertion in *PML* at intron 3 (bcr3 region). The insertion is shown as a blue bar in the SV events track (**C**). Genome browser view using the BAS software shows the missing alignment of OGM molecules on the *RARA* gene highlighted by the red arrows. (**D**). The low coverage of OGM molecules from exon 1 and 2 and the breakpoint in intron 2 of the *RARA* gene confirms the missing alignment of molecules using the VIA software. (**E**). The possible S-isoform with type A translocation/fusion was constructed based on the available breakpoints and intron 3 involvement of the *PML* gene.

OGM analysis using BAS and VIA revealed an insertion of a ~62 kb segment in the *PML* gene in intron 3 (bcr3 transcript) at the breakpoints 73995446 and 74023755 of 15q24.1 (Figure 3A,B). Further analysis in the 17q21.2 region revealed missing alignment and low OGM molecule coverage in the *RARA* gene from exon 1 and 2 with the breakpoint 40335716 in intron 2 (Figure 3C,D). These missing molecules matched the 62 Kb insertion in the *PML* gene. Based on the OGM data, *PML/RARA* breakpoints were identified as the S-isoform (bcr3), confirming the RT-PCR results (Figure 3E).

Fortunately, despite negative FISH results, the patient had been started on ATRA/ATO therapy early based on the classical immunomorphology. Presently, the patient is in remission and undergoing consolidation therapy.

### 3.5. Literature Review

A total of 71 articles were retrieved, of which 29 articles were selected for further evaluation based on preliminary abstract review. Of these, 17 articles described single case reports of APL with cryptic *PML::RARA* rearrangements and are summarized in Table 1. The remaining reports are described below.

## 4. Discussion

While APL can be suspected based on clinical presentation and pathology evaluation [46], the definitive diagnosis requires the demonstration of the cytogenetic hallmark, t(15;17)(q24;q21) translocation by G-banding or targeted FISH. The chimeric *PML::RARA* oncoprotein, along with secondarily acquired genetic aberrations (e.g., mutations in *FLT3* ITD, *WT1*, *NRAS*, or *KRAS*) [47,48], impairs myeloid differentiation and drives leukemogenesis. We describe the case of a 36-year-old male who presented with signs of clinical coagulopathy. Although pathology evaluation in our patient suggested a diagnosis of APL, stat FISH and karyotype analysis were negative for t(15;17)(q24;q21). Concomitant RT-PCR revealed a cryptic *PML::RARA* gene fusion. Fortunately, the patient had been initiated on ATRA/ATO and demonstrated clinical improvement. Accordingly, we performed a systematic literature review to understand the prevalence, diagnosis, and prognosis of APL with cryptic *PML::RARA* translocations.

Several individual published reports of APL with little-to-no cytogenetic evidence of t(15;17) but with nearly identical clinical presentations and morphologies have been reported in the literature, with RT-PCR later revealing a cryptic *PML::RARA* fusion (Table 1) [31,32,33]. In an institutional analysis over an 18.5-year timeframe, Gagnon et al. [9] found the majority of APL cases (87% [723/831]) to possess balanced *PML::RARA* translocations by chromosomal banding analysis and *PML::RARA* dual-color, dual-fusion FISH (Abbott).Of the remaining 13% of patients, only 0.7% (6/831) were found to harbor a cryptic *PML::RARA* gene fusion, confirmed by retrospective metaphase FISH in two cases [9]. Goldschmidt et al. describe a case of APL with a cryptic *PML::RARA* gene rearrangement, in which subsequent metaphase FISH revealed an interstitial insertion of *RARA* into *PML* [38]. Burns et al. present a similar case but with an insertion of *PML* into *RARA* [33]. Interestingly, in a case of APL with a cryptic *PML::RARA* translocation, identified by RT-PCR, Koshy et al. detected a 49-kilobase duplication of 15q24.1 by SNP microarray, which likely inserted into chromosome 19 or 20; they hypothesize that the *RARA* insert in this case was too small to detect using FISH or microarray [39]. Other methods of confirmation include sequencing (both Sanger and next-generation).

Likewise, atypical karyotypic findings are present in nearly 52% of cases of cryptically rearranged APL [40]. Chromosomal aberrations reported in conjunction with cryptic *PML::RARA* rearrangements include trisomy 8 (most common) [7], trisomy 9 [38], i(5p) [38], complex karyotypes [37,44], and structural rearrangements involving chromosome 17 [4] (e.g., isochromosome 17q) [43,44,49,50]. Few studies suggest the presence of i(17q) in such cases to confer a worse prognosis, with short complete response durations and high rates of relapse [44]. Similarly, rearrangements involving chromosomes 3, 7, 9, 11, 17, and 22 have also been reported in association with cases of FISH-negative APL [41]. Of note, the presence of structural karyotypes does not seem to affect treatment response to ATRA/ATO [37].

Importantly, all patients with APL resulting from a cryptic t(15;17) achieved remission following treatment with ATRA/ATO [4,31,32]. Greenfield et al. compared five patients with cryptically rearranged APL to eight patients with APL resulting from the reciprocal t(15;17)(q24.1;q21.2) and found no significant difference in disease behavior or prognosis [51]. Furthermore, previous reports observed the bcr3 isoform to be more frequent in cases of cryptic *PML::RARA*-rearranged APL (61% of cases) [7], as was observed in our case. Although this isoform has been associated with leukocytosis and a poorer prognosis, further studies are needed to understand the association, if any [8,38,40].

Given the aggressive coagulopathy associated with APL (85% of cases) [7], it is imperative that cryptic *PML::RARA* translocations are identified early to initiate ATRA/ATO given the resistance of APL to standard induction chemotherapy regimens. FISH is an invaluable tool to detect the classical t(15;17) translocation with a resolution of nearly 200 kb; however, cryptic cases may be missed due to submicroscopic insertions unable to be visualized by fluorescent microscopy [52]. Although smaller custom FISH probes can be applied [52], alternative testing modalities, such as RT-PCR, OGM, or sequencing, may be required to establish a diagnosis of APL [8,31,32,39,42,53].

In our case, initial direct interphase FISH (without culture) using both the Vysis dual color dual fusion t(15;17) and the breakapart *RARA* probes showed normal results. Follow-up karyotype and repeat FISH were normal. Based on the positive RT-PCR findings, retrospective interphase/metaphase FISH using the CytoCell *PML/RARA* dual-color dual-fusion probe set revealed a *PML::RARA* fusion with prior knowledge of RT-PCR positivity. Routinely, the cryptic *PML::RARA* fusion signal is often difficult to detect even using the CytoCell probe. Thus, both chromosome analysis and FISH would be less efficient or incapable of detecting cryptic *PML::RARA* rearrangements.

RT-PCR testing in our case confirmed a cryptic *PML::RARA* rearrangement despite normal chromosome and FISH findings. This provides evidence that RT-PCR offers significant advantages over standard cytogenetic/FISH testing for detecting cryptic *PML-RARA* fusions in APL. It is also important to note that RT-PCR provides high sensitivity capability to identify MRD, and detection of low levels transcripts, which are crucial for monitoring treatment response. It also provides quantitative analysis, enabling the precise measurement of *PML-RARA* transcript levels and differentiation between various fusion isoforms [17,18,19,20]. Seldom, RT-PCR may also be negative in the case of an atypical *PML::RARA* rearrangement with a new breakpoint in *PML* that is not readily identifiable using routine primers [38]. Finally, both conventional cytogenetics and RT-PCR may fail to yield a positive result, such as cases with a submicroscopic deletion of 3′ *RARA* or cases with no *RARA* rearrangement [38,54,55].

Finally, OGM analysis in our case provided a precise and comprehensive characterization of the *PML/RARA* rearrangement, which were undetected by karyotype and the standard *PML-RARA* FISH probes. OGM revealed a cryptic insertion of ~62 kb of the *RARA* gene into the *PML* gene region, within intron 3. As shown in Figure 3, the missing molecule alignment on the *RARA* gene with the breakpoint in intron 2 is inserted within the *PML* region, confirming the RT-PCR finding of a short isoform of *PML/RARA* (bcr3 transcript). Recent reports suggest that OGM is an effective alternative to detect cryptic and complex *PML/RARA* rearrangements, similar to our case, that may be undetected by cytogenetic/FISH testing in APL patients [56,57]. In comparison to other cytogenetic testing modalities, OGM can detect copy number variants, insertions, inversions, balanced and unbalanced translocations using a single platform [52]. It offers high-resolution, genome-wide, unbiased analysis, enabling precise characterization of cryptic rearrangements and fusion breakpoints. With an easy workflow and a fast turnaround time of approximately four days, OGM simplifies the process and is highly suitable for routine clinical use, requiring no cell culture and enabling quicker diagnosis and treatment decisions [23,24,25,26].

In summary, a high degree of clinical suspicion and prompt recognition are required in suspected cases of APL for immediate initiation of ATRA/ATO [7,58]. Although rare, cryptically rearranged APL poses a diagnostic challenge and may require alternative, parallel testing modalities to establish the diagnosis [52,58]. Together, RT-PCR and OGM address the limitations of conventional cytogenetic methods, providing a synergistic approach that enhances diagnostic accuracy, particularly for cryptic and complex genomic alterations, thereby enabling timely and precise therapeutic interventions in APL.

## Figures and Tables

**Table 1:** Previously reported single cases of APL with cryptic t(15;17).

No.		Age/Sex	Clinical Presentation	Microscopic Findings	Flow Cytometry Expression	Karyotype	Interphase FISH	Mutational Analysis	RT-PCR Results	Confirmatory Testing
1	[31]	12/F	Multiple ecchymoses	Leukocytosis Abnormal promyelocytes with bilobed nuclei and cytoplasmic granules Anemia Thrombocytopenia	MPO, CD34 CD123, CD64, CD33, CD117, CD9, HLA-DR and CD2, while negative for other markers	46, XX	Abbott Molecular LSI PML/RARA dual-color dual-fusion translocation probe: Negative Cytocell (Cambridge, UK) positive for PML::RARA fusion	FLT3-ITD	bcr3-*PML/RARA* transcript	Sanger sequencing: in-frame fusion of *PML* exon 3 and *RARA* exon 3
2	[32]	17/M	Gum bleeding, multiple ecchymoses, abdominal pain, and fever	Leukocytosis Abnormal promyelocytes with bilobed nuclei and cytoplasmic granules	CD117, CD45 (dim), CD13, CD33, CD15 (weak), and CD64 while negative for HLA-DR, CD34, and other markers	46, XY	Negative	FLT3-ITD	bcr1-*PML/RARA* transcript	Sequencing: in-frame fusion of *PML* exon 6 and *RARA* exon 3
3	[33]	23/F	Epistaxis and easy bruising	Leukocytosis Abnormal myeloblasts/promyelocytes with ovoid nuclei and cytoplasmic granules (rare Auer rods) Anemia Thrombocytopenia	CD13, CD33 (partial), CD56 (partial, dim), CD64, and MPO, while negative for HLA-DR and CD34	46, XX,+8	Abbott Molecular LSI *PML/RARA* dual-color dual-fusion translocation probe: Negative	Not mentioned	Cryptic *PML::RARA* fusion	Metaphase FISH: interstitial insertion of *PML* into the *RARA* gene
4	[4]	8/M	Bruising and bleeding gums	Leukocytosis Blasts and abnormal promyelocytes with large irregularly folded or bi-lobed nuclei and abnormal granulation (rare Auer rods) Anemia Thrombocytopenia	CD13, CD33, CD117, CD123, and CD45, while negative for other markers	46,XY,der(17)ins(17; 15) (q21;q24q24)?del(17)(p11.2)add(17)(q21)	MetaSystems, Germany dual color dual fusion PML-RARA probe: single fusion signal and 2 copies of *PML* and *RARA*; second expected reciprocal fusion signal not present, and one each of the *PML* and *RARA* signals was of diminished intensity	FLT3-ITD	bcr3-*PML/RARA* transcript and a faint ∼ 350-bp product of unknown origin	Metaphase FISH (using *RARA* break-apart probe [Abbott Molecular, USA], 15q11.2 control locus [RP11-160D9 from Australasian Genome Research Facility, Melbourne, Australia], subtelomere clones for chromosome 15q [GS-154P1], 17p [cosmid 2111b1], and 17q [PAC GS-362K4], and *NF1* within chromosome band 17q11.2): single fusion with diminished *RARA* signals on the derivative chromosome 17; i.e., der(17) 850K SNP chromosome microarray: no clinically relevant chromosome copy number abnormality across the tumor genome
5	[34]	61/F	Fatigue and easy bruising	Leukocytosis Blasts/promyelocytes 40% Anemia Thrombocytopenia	Dim CD45, CD13, CD33,CD117, variable CD34, and lacking HLA-DR	46, XX, +8 (17/20 cells)	Peripheral blood: variant abnormal signal pattern with 1fusion (1F1O2G) in 52.5% of the nuclei Bone marrow: variant abnormal signal pattern with 1fusion (1F1O2G) in 42% of the nuclei	Not mentioned	Cryptic *PML::RARA* fusion without reciprocal *RARA-PML* fusion transcripts in either the diagnostic or follow-up samples	Metaphase FISH: Non-reciprocal translocation with the fusion signal on chromosome 15 and absence of the fusion signal on chromosome 17 Metaphase FISH using whole chromosome paint: *RARA*(green) signal on chromosome15, without the corresponding *PML* (orange) signal on chromosome 17, demonstrating an insertion
6	[35]	17/M	Seizure (with recent history of nausea, blood-tinged vomiting, lethargy, and right-sided weakness)	Leukocytosis Blasts/promyelocytes 83% Anemia Thrombocytopenia	CD2 (partial), CD4 (partial), CD13, CD33, CD38, CD45, CD64, CD117 (partial), HLA-DR (small subset), and MPO (bright), while negative for other markers	46, XY	Negative	Not mentioned	Cryptic *PML::RARA* fusion	Not performed
7	[36]	66/M	Petechiae and bruises	Leukopenia Blasts/promyelocytes 68% (irregular nuclear shapes, misty nucleoli) Anemia Thrombocytopenia	CD34, CD7, CD13, CD33, CD117, CD38, HLA-DR and MPO, while negative for other markers	46, XY	Negative	Biallelic CEBPA mutation	bcr2-*PML/RARA* transcript (nested RT-PCR)	Not performed
8	[37]	62/M	Pharyngalgia, fatigue, and gum bleeding	Anemia Leukopenia Hypercellular bone marrow with abnormal promyelocytes	CD117, CD33, myeloperoxidase (MPO), CD13, CD58, CD38, and CD81	46,XY, add(11) (p15), and ?t(13;20)(q12;q11.2)	Atypical PML::RARA fusion signal in 91% of nuclei	FLT3, WT1, and KRAS mutations	Major PML/RARα transcript harbored the three type breakpoints	Not performed
9	[8]	18/M	Hematuria and hematochezia	Leukocytosis Blasts/promyelocytes 84% Anemia Thrombocytopenia	CD13, CD33, CD45, and CD117 and negative for HLA-DR and CD34	46,XY	Negative	Not mentioned	Three PML::RARA fusion transcripts: bcr2, bcr1, and novel transcript (exon 4 of PML and exon 3 of RARA)	Long-distance DNA-PCR: rearrangement between PML (intron 6) and RARA (intron 2)
10	[38]	52/F	Bleeding tendency	Anemia Leukopenia Thrombocytopenia	Strongly positive for CD33 and CD13 while negative for HLA-DR	47,XX,zi(5)(p10)[20]/48,idem,z9[2]/46,XX[6]	Negative	Not mentioned	Bcr1-PML/RARa transcript	Metaphase FISH: interstitial insertion of RARa gene into PML gene (low signal retrospectively identified on interphase FISH)
11	[39]	29/M	Progressive fatigue Bruises	Pancytopenia 26% abnormal promyelocytes	Positive for CD117, CD33, and CD13 but negative for HLA-DR and CD34	46,XY	Negative with a small PML signal present on another chromosome (20% of cells)	Not mentioned	Positive for *PML::RARA* transcript	Sanger sequencing across the PML-RARA breakpoint demonstrated a BCR1-type fusion. Whole genome SNP microarray: intragenic duplication of PML on chromosome 15q24.1 (30% of cells).
12	[40]	54/M	DVT	Pancytopenia 77% abnormal promyelocytes in the BM	Positive for MPO and CD117 while negative for CD34 and HLA-DR	46,XY	Negative	*FLT3* p.D835Y variant	Bcr1-PML/RARA transcript	Not performed
13	[41]	68/F	Dizziness Fatigue Acute on chronic PE	Pancytopenia 70% abnormal promyelocytes/blasts	Not mentioned	46, XX	Two copies of chromosome 15, but absence of the reciprocal translocation on the two copies of chromosome 17	Not mentioned	Positive for bcr3-*PML::RARA* transcript	Metaphase FISH: insertion of a RARA segment into chromosome 15 at the location of PML. Whole-genome sequencing: complex t(15;17) with a possible intrachromosomal rearrangement of chromosome 15.
14	[42]	57/F	Bruising Gingival bleeding	Anemia Neutropenia Thrombocytopenia Abnormal promyelocytes/blasts	Positive for CD13, CD33, CD34 (partial), CD117, MPO, and aberrant partial CD2 expression	46, XX	Negative	Not mentioned	PML-RARA fusion in 53% of cells	Mate-pair sequencing: cryptic insertional translocation resulting in PML-RARA fusion with breakpoints located within intron 6 of PML and intron 2 of RARA.
15	[43]	53/F	Not mentioned	Not mentioned	Not mentioned	46XX, iso(17)(q11)	Using the Cytocell probe: negative	Not mentioned	Major PML−RARA transcript harbored the BCR-1 breakpoint. BCR-2 and BCR-3 were demonstrated as spliced variants.	FISH using the Vysis probe: several clones detected
16	[44]	21/M	Melena Bleeding tendency	Not mentioned	Not mentioned	Complex karyotype with isochromosome 17q	Two PML/RARA fusion signals	Not mentioned	Not mentioned	Not performed
17	[45]	73/F	Diagnosed incidentally (pre-operatively)	Anemia Thrombocytopenia Abnormal promyelocytes/blasts	Positive for CD13, CD33, MPO, CD2 and CD9, while negative for other markers	46,XX	Two fusion signals on the two copies of chromosome 15, but absence of the reciprocal on the two copies of chromosome 17	Not mentioned	Bcr3/short form PML-RARA fusion transcript	Metaphase FISH: two PML/RARA fusion signals (one on each copy of chromosome 15, and two normal RARA signals on the two copies of chromosome 17), raising the possibility of uniparental isodisomy.

## Data Availability

The original contributions presented in the study are included in the article, further inquiries can be directed to the corresponding author.

## References

[1] Jimenez J.J., Chale R.S., Abad A.C., Schally A.V. (2020). Acute promyelocytic leukemia (APL): A review of the literature. Oncotarget.

[2] Alaggio R.A.C., Anagnostopoulos I., Attygalle A.D., Araujo I.B.O., Berti E., Bhagat G., Borges A.M., Boyer D., Calaminici M., Chadburn A. (2022). The 5th edition of the World Health Organization Classification of Haematolymphoid Tumours: Lymphoid Neoplasms. Leukemia.

[3] Stone M., Lilley C.M., Tang G., Loghavi S., Mirza K.M. (2023). Phenotypic clues that predict underlying cytogenetic/genetic abnormalities in myeloid malignancies: A contemporary review. Cytopathology.

[4] El-Hajj Ghaoui R., St Heaps L., Hung D., Nagabushan S., Harris C., Mirochnik O., Sharma P., Kellie S.J., Wright D.C. (2017). A Paediatric Acute Promyelocytic Leukaemia Patient Harbouring a Cryptic PML-RARA Insertion due to a Complex Structural Chromosome 17 Rearrangement. Cytogenet. Genome Res..

[5] Arana Rosainz M.J., Nguyen N., Wahed A., Lelenwa L.C., Aakash N., Schaefer K., Rios A., Kanaan Z., Chen L. (2021). Acute myeloid leukemia with mutated *NPM1* mimics acute promyelocytic leukemia presentation. Int. J. Lab. Hematol..

[6] Mason E.F., Kuo F.C., Hasserjian R.P., Seegmiller A.C., Pozdnyakova O. (2018). A distinct immunophenotype identifies a subset of *NPM1*-mutated AML with *TET2* or *IDH1/2* mutations and improved outcome. Am. J. Hematol..

[7] Rashidi A., Fisher S.I. (2015). FISH-negative, cytogenetically cryptic acute promyelocytic leukemia. Blood Cancer J..

[8] Kim M.J., Cho S.Y., Kim M.H., Lee J.J., Kang S.Y., Cho E.H., Huh J., Yoon H.J., Park T.S., Lee W.I. (2010). FISH-negative cryptic PML-RARA rearrangement detected by long-distance polymerase chain reaction and sequencing analyses: A case study and review of the literature. Cancer Genet. Cytogenet..

[9] Gagnon M.F., Berg H.E., Meyer R.G., Sukov W.R., Van Dyke D.L., Jenkins R.B., Greipp P.T., Thorland E.C., Hoppman N.L., Xu X. (2022). Typical, atypical and cryptic t(15;17)(q24;q21) (PML::RARA) observed in acute promyelocytic leukemia: A retrospective review of 831 patients with concurrent chromosome and PML::RARA dual-color dual-fusion FISH studies. Genes Chromosomes Cancer.

[10] Singh M.K., Parihar M., Arora N., Mishra D.K., Bhave S.J., Chandy M. (2019). Diagnosis of variant RARA translocation using standard dual-color dual-fusion PML/RARA FISH probes: An illustrative report. Hematol. Oncol. Stem Cell Ther..

[11] Catalano A., Dawson M.A., Somana K., Opat S., Schwarer A., Campbell L.J., Iland H. (2007). The PRKAR1A gene is fused to RARA in a new variant acute promyelocytic leukemia. Blood.

[12] Osumi T., Watanabe A., Okamura K., Nakabayashi K., Yoshida M., Tsujimoto S.I., Uchiyama M., Takahashi H., Tomizawa D., Hata K. (2019). Acute promyelocytic leukemia with a cryptic insertion of RARA into TBL1XR1. Genes. Chromosomes Cancer.

[13] Pardo Gambarte L., Franganillo Suarez A., Cornago Navascues J., Soto de Ozaeta C., Blas Lopez C., Atance Pasarisas M., Salgado Sanchez R.N., Serrano Del Castillo C., Mata Serna R., Velasco Rodriguez D. (2022). ZBTB16-RARalpha-Positive Atypical Promyelocytic Leukemia: A Case Report. Medicina.

[14] Wang Y., Rui Y., Shen Y., Li J., Liu P., Lu Q., Fang Y. (2021). Myeloid Sarcoma Type of Acute Promyelocytic Leukemia With a Cryptic Insertion of RARA Into FIP1L1: The Clinical Utility of NGS and Bioinformatic Analyses. Front. Oncol..

[15] Peterson J.F., He R.R., Nayer H., Cuevo R.S., Smadbeck J.B., Vasmatzis G., Greipp P.T., Ketterling R.P., Hoppman N.L., Baughn L.B. (2019). Characterization of a rarely reported STAT5B/RARA gene fusion in a young adult with newly diagnosed acute promyelocytic leukemia with resistance to ATRA therapy. Cancer Genet..

[16] Yao L., Wen L., Wang N., Liu T., Xu Y., Ruan C., Wu D., Chen S. (2018). Identification of novel recurrent STAT3-RARA fusions in acute promyelocytic leukemia lacking t(15;17)(q22;q12)/PML-RARA. Blood.

[17] Cassinat B., Zassadowski F., Balitrand N., Barbey C., Rain J., Fenaux P., Degos L., Vidaud M., Chomienne C. (2000). Quantitation of minimal residual disease in acute promyelocytic leukemia patients with t(15;17) translocation using real-time RT-PCR. Leukemia.

[18] Tobal K., Moore H., Macheta M., Yin J.L. (2001). Monitoring minimal residual disease and predicting relapse in APL by quantitating PML-RARα transcripts with a sensitive competitive RT-PCR method. Leukemia.

[19] Visani G., Buonamici S., Malagola M., Isidori A., Piccaluga P., Martinelli G., Ottaviani E., Grafone T., Baccarani M., Tura S. (2001). Pulsed ATRA as single therapy restores long-term remission in PML-RARα-positive acute promyelocytic leukemia patients: Real time quantification of minimal residual disease. A pilot study. Leukemia.

[20] Gallagher R.E., Yeap B.Y., Bi W., Livak K.J., Beaubier N., Rao S., Bloomfield C.D., Appelbaum F.R., Tallman M.S., Slack J.L. (2003). Quantitative real-time RT-PCR analysis of PML-RARalpha mRNA in acute promyelocytic leukemia: Assessment of prognostic significance in adult patients from intergroup protocol 0129. Blood.

[21] Prabhash K., Polampalli S., Choughule A., Amare P., Baisane C., Kabre S., Mahadik S., Shinde S., Nair R., Banavali S. (2011). Role of RT-PCR and FISH in diagnosis and monitoring of acute promyelocytic leukemia. Indian J. Cancer.

[22] Tobal K., Yin J.L. (1998). RT-PCR method with increased sensitivity shows persistence of PML-RARA fusion transcripts in patients in long-term remission of APL. Leukemia.

[23] Smith A.C., Neveling K., Kanagal-Shamanna R. (2022). Optical genome mapping for structural variation analysis in hematologic malignancies. Am. J. Hematol..

[24] Neveling K., Mantere T., Vermeulen S., Oorsprong M., van Beek R., Kater-Baats E., Pauper M., van der Zande G., Smeets D., Weghuis D.O. (2021). Next-generation cytogenetics: Comprehensive assessment of 52 hematological malignancy genomes by optical genome mapping. Am. J. Hum. Genet..

[25] Valkama A., Vorimo S., Kumpula T.A., Räsänen H., Savolainen E.-R., Pylkäs K., Mantere T. (2023). Optical Genome Mapping as an Alternative to FISH-Based Cytogenetic Assessment in Chronic Lymphocytic Leukemia. Cancers.

[26] Sahajpal N.S., Mondal A.K., Tvrdik T., Hauenstein J., Shi H., Deeb K.K., Saxe D., Hastie A.R., Chaubey A., Savage N.M. (2022). Clinical Validation and Diagnostic Utility of Optical Genome Mapping for Enhanced Cytogenomic Analysis of Hematological Neoplasms. J. Mol. Diagn..

[27] Nilius-Eliliwi V., Gerding W.M., Schroers R., Nguyen H.P., Vangala D.B. (2023). Optical Genome Mapping for Cytogenetic Diagnostics in AML. Cancers.

[28] Seabright M. (1971). A rapid banding technique for human chromosomes. Lancet.

[29] Liehr T. (2020). An International System for Human Cytogenomic Nomenclature.

[30] Pang A.W.C., Kosco K., Sahajpal N.S., Sridhar A., Hauenstein J., Clifford B., Estabrook J., Chitsazan A.D., Sahoo T., Iqbal A. (2023). Analytic Validation of Optical Genome Mapping in Hematological Malignancies. Biomedicines.

[31] Avgerinou G., Katsibardi K., Filippidou M., Tzanoudaki M., Papadhimitriou S.I., Kattamis A. (2020). Cytogenetically cryptic and fish negative PML/RARA rearrangement in acute promyelocytic leukemia detected by RT-PCR. Leuk. Lymphoma.

[32] Blanco E.M., Curry C.V., Lu X.Y., Sarabia S.F., Redell M.S., Lopez-Terrada D.H., Roy A. (2014). Cytogenetically cryptic and FISH-negative PML/RARA rearrangement in acute promyelocytic leukemia detected only by PCR: An exceedingly rare phenomenon. Cancer Genet..

[33] Burns T.F., Loo E.Y., Bengtson E.M., Bao L. (2019). Cytogenetically cryptic insertion of PML segment into RARA on chromosome 17q resulting PML-RARA fusion in acute promyelocytic leukemia. Ann. Hematol..

[34] Fan H., Ortega V., Fanasch H.M., Wang Y., Holder K.N., Higgins R.A., Mendiola C., Mohamed G., Vadlamudi K., Velagaleti G. (2014). PML-RARA fusion resulting from a cryptic insertion of *RARA* gene into *PML* gene without the reciprocal RARA-PML fusion: Clinical, cytogenetic, and molecular characterization and prognosis. Eur. J. Haematol..

[35] Mai B., Liang C., Nguyen A., Wahed A., Chen L. (2020). Cryptic Acute Promyelocytic Leukemia (APL) Presenting as Seizures in an Ad-olescent. Ann. Clin. Lab. Sci..

[36] Zhang Z., Xu Y., Jiang M., Kong F., Chen Z., Liu S., Li F. (2020). Identification of a new cryptic PML-RARα fusion gene without t(15;17) and biallelic CEBPA mutation in a case of acute promyelocytic leukemia: A case detected only by RT-PCR but not cytogenetics and FISH. Cancer Biol. Ther..

[37] Gu S., Zi J., Ma J., Ge Z. (2021). Cryptic t(15;17) acute promyelocytic leukemia with a karyotype of add(11)(p15) and t(13,20)—A case report with a literature review. Bosn. J. Basic Med. Sci..

[38] Goldschmidt N., Yehuda-Gafni O., Abeliovich D., Slyusarevsky E., Rund D. (2010). Interstitial insertion of *RARα* gene into*PML*gene in a patient with acute promyelocytic leukemia (APL) lacking the classic t(15;17). Hematology.

[39] Koshy J., Qian Y.-W., Bhagwath G., Willis M., Kelley T.W., Papenhausen P. (2012). Microarray, gene sequencing, and reverse transcriptase–polymerase chain reaction analyses of a cryptic PML-RARA translocation. Cancer Genet..

[40] Karlin K., Bryke C., Dias A., Michaels P. (2022). Cytogenetically cryptic PML::RARA fusion in acute promyelocytic leukemia: Testing strategies in the modern era. Leuk. Res. Rep..

[41] Mahmud W., Brown R., Buckingham L., Tira A., Katz D.A. (2020). Cryptic partial insertion of the *RARA* gene into the *PML* gene without reciprocal *RARA-PML* fusion: A case report and review of literature. Acta Oncol..

[42] Schultz M.J., Blackburn P.R., Cogbill C.H., Pitel B.A., Smadbeck J.B., Johnson S.H., Vasmatzis G., Rech K.L., Sukov W.R., Greipp P.T. (2020). Characterization of a cryptic *PML-RARA* fusion by mate-pair sequencing in a case of acute promyelocytic leukemia with a normal karyotype and negative *RARA* FISH studies. Leuk. Lymphoma.

[43] Shepshelovich D., Oniashvili N., Parnes D., Klein A., Muchtar E., Yeshaya J., Aviram A., Rabizadeh E., Raanani P. (2015). Acute promyelocytic leukemia with isochromosome 17q and cryptic PML-RARA successfully treated with all-trans retinoic acid and arsenic trioxide. Cancer Genet..

[44] Tang Y., Wang Y., Hu L., Meng F., Xu D., Wan K., Huang L., Li C., Zhou J. (2015). Acute promyelocytic leukemia with cryptic t(15;17) on isochromosome 17: A case report and review of literature. Int. J. Clin. Exp. Pathol..

[45] Venci A., Mazza R., Spinelli O., Di Schiena L., Bettio D. (2017). Acute promyelocytic leukemia with a cryptic insertion of RARA into PML on chromosome 15 due to uniparental isodisomy: A case report. Oncol. Lett..

[46] Liu G., Liu L., Di Bartolo D., Li K.Y., Li X. (2022). Acute Promyelocytic Leukemia with Rare Genetic Aberrations: A Report of Three Cases. Genes.

[47] Fasan A., Haferlach C., Perglerovà K., Kern W., Haferlach T. (2017). Molecular landscape of acute promyelocytic leukemia at diagnosis and relapse. Haematologica.

[48] Madan V., Shyamsunder P., Han L., Mayakonda A., Nagata Y., Sundaresan J., Kanojia D., Yoshida K., Ganesan S., Hattori N. (2016). Comprehensive mutational analysis of primary and relapse acute promyelocytic leukemia. Leukemia.

[49] Cervera J., Montesinos P., Hernández-Rivas J.M., Calasanz M.J., Aventín A., Ferro M.T., Luño E., Sánchez J., Vellenga E., Rayón C. (2010). Additional chromosome abnormalities in patients with acute promyelocytic leukemia treated with all-trans retinoic acid and chemotherapy. Haematologica.

[50] Kim M., Lim J., Kim Y., Han K., Lee D.H., Chung N.G., Cho B., Kim H.K., Eom K.S., Min C.-K. (2008). The genetic characterization of acute promyelocytic leukemia with cryptic t(15;17) including a new recurrent additional cytogenetic abnormality i(17)(q10). Leukemia.

[51] Greenfield G., Michail O., Merron B., McGimpsey J., Catherwood M., McMullin M.F. (2019). Acute promyelocytic leukaemia (APML) with cryptic *PML*-*RARA* fusion has a clinical course comparable to classical APML with t(15;17)(q24.1;q21.2) translocation. Br. J. Haematol..

[52] Mohebnasab M., Li P., Hong B., Dunlap J., Traer E., Fan G., Press R.D., Moore S.R., Xie W. (2023). Cytogenetically Cryptic Acute Promyelocytic Leukemia: A Diagnostic Challenge. Int. J. Mol. Sci..

[53] Campbell L.J., Oei P., Brookwell R., Shortt J., Eaddy N., Ng A., Chew E., Browett P. (2013). FISH detection of *PML*-*RARA* fusion in ins(15;17) acute promyelocytic leukaemia depends on probe size. BioMed. Res. Int..

[54] Grimwade D., Biondi A., Mozziconacci M.J., Hagemeijer A., Berger R., Neat M., Howe K., Dastugue N., Jansen J., Radford-Weiss I. (2000). Characterization of acute promyelocytic leukemia cases lacking the classic t(15;17): Results of the European Working Party. Groupe Francais de Cytogenetique Hematologique, Groupe de Francais d’Hematologie Cellulaire, UK Cancer Cytogenetics Group and BIOMED 1 European Community-Concerted Action “Molecular Cytogenetic Diagnosis in Haematological Malignancies”. Blood.

[55] Han Y., Xue Y., Zhang J., Pan J., Wu Y., Bai S. (2009). Y-chromosome loss as the sole karyotypic anomaly with 3’RARalpha submicroscopic deletion in a case of M3r subtype of acute promyelocytic leukemia. Leuk. Res..

[56] Klausner M., Stinnett V., Ghabrial J., Morsberger L., DeMetrick N., Long P., Zhu J., Smith K., James T., Adams E. (2024). Optical Genome Mapping Reveals Complex and Cryptic Rearrangement Involving *PML*::*RARA* Fusion in Acute Promyelocytic Leukemia. Genes.

[57] Sathyanarayana S.H., Bickford M.A., Smuliac N.A., Tonseth K.A., Murad F., Bao J., Steinmetz H.B., Sullivan M.R., Kaur P., Karrs J.X. (2024). 19. High resolution cytogenomic analysis reveals characterizing abnormalities in APL-like leukemia. Cancer Genet..

[58] Singh J., Facey A., O’malley F., Ryland G.L., Blombery P., Gregory G.P. (2021). Cryptic molecular lesion in acute promyelocytic leukemia with negative initial FISH. Leuk. Lymphoma.

